# Immunogenicity and safety of the Southern Hemisphere 2015 formulation of Vaxigrip®

**DOI:** 10.1080/21645515.2017.1363944

**Published:** 2017-09-22

**Authors:** Mathilde Latreille-Barbier, Regine Rouzier, Beatrice Astruc, Nathalie Lavis, Yves Donazzolo

**Affiliations:** a Eurofins OPTIMED, Gières, France; b Centre CAP, Centre Médical Odysseum, Montpellier, France; c Biotrial, Rennes, France; d Sanofi Pasteur, Lyon, France

**Keywords:** immunogenicity, safety, Southern Hemisphere, trivalent inactivated influenza vaccine

## Abstract

An inactivated split-virion trivalent influenza vaccine (IIV3; Vaxigrip®, Sanofi Pasteur) has been available globally since 1968. Here, we describe the results of an open-label, post-licensure trial (EudraCT no. 2014-005078-12) to confirm the immunogenicity and safety of the Southern Hemisphere 2015 formulation of IIV3. Adults 18–60 years of age and > 60 years of age (60 per age group) received a single 0.5-ml intramuscular injection of IIV3. Between baseline and day 21 after vaccination, hemagglutination inhibition (HAI) titers for each strain in IIV3 increased, on average, by at least 11-fold for younger adults and at least 5-fold for older adults. After vaccination, 89%–100% of the younger adult participants and 90%–98% of the older adult participants attained seroprotection (HAI titer ≥ 40) for each strain. Also, 66%–81% of younger adults and 45%–63% of older adults seroconverted or had a significant increase in HAI titer for each strain. For both age groups, these post-vaccination immune responses exceeded the criteria of the Committee for Human Medicinal Products former Note for Guidance for influenza vaccines. No serious adverse events were reported, and no new safety signals were detected. In conclusion, this study confirmed that the Southern Hemisphere 2015 formulation of IIV3 was well tolerated, highly immunogenic, and met the criteria for influenza vaccine efficacy and safety.

Since 1999, the *Sistema Único de Saúde* of Brazil has carried out annual influenza vaccine campaigns.^1^ Target populations include children between 6 months and 5 years of age, adults ≥ 60 years of age, pregnant women, women within 45 days after giving birth, healthcare professionals, individuals with chronic respiratory diseases or with transplants, prisoners and people working in the correctional system, and the indigenous population.^2^ The influenza vaccine has been provided free of charge, usually beginning in March or April,^1^ which coincides with the beginning of the influenza season in the northern equatorial region.^3^ Coverage of targeted populations in Brazil has been 60%–90%.^1, 4^

An inactivated split-virion trivalent influenza vaccine (IIV3; Vaxigrip®, Sanofi Pasteur) has been available globally since 1968 and in Brazil since 1984.^5^ In compliance with World Health Organization recommendations for seasonal influenza vaccines, Vaxigrip contains hemagglutinin from two influenza A strains (H1N1 and H3N2) and one B strain. The vaccine has been shown to reduce the incidence of influenza infection, decrease workplace absenteeism, and decrease hospitalization and mortality in older adults and other at-risk populations.^6^ Long-term experience has shown that the vaccine is well tolerated^7^ and, compared with no vaccination, does not increase the rate of clinically important, medically attended events.^8,9^

Here, we describe the results of an open-label, post-licensure trial (EudraCT no. 2014-005078-12) requested by the Brazilian health authorities (*Agência Nacional de Vigilância Sanitária*), to confirm the immunogenicity and safety of the Southern Hemisphere 2015 formulation of IIV3. The vaccine was evaluated according to the European Medicine Agency's Committee for Medicinal Products for Human Use (CHMP) former Note for Guidance.^10^ New guidelines became available in 2016,^11^ after this study was completed.

The study was conducted at three sites in France and included 60 younger adults (18–60 years of age) and 60 older adults (> 60 years of age) to meet the minimum of 50 participants per group recommended in the former CHMP Note for Guidance (Table 1). In both age groups, approximately two-thirds of the participants were female. Mean ages were 37.8 years in the younger adult group and 67.3 years in the older adult group. All participants, except for one younger adult who withdrew consent, received a single 0.5-ml intramuscular injection of the licensed 2015 Southern Hemisphere formulation of IIV3.

**Table 1.** Participant characteristics and disposition.

Disposition/characteristic	18–60 y	>60 y
Disposition, n		
Enrolled	60	60
Withdrew consent before vaccination	1	0
Vaccinated	59	60
Completed the study	59	60
Characteristic		
Age (y), mean ± standard deviation [range]	37.8 ± 13.3 [19.0–60.0]	67.3 ± 5.0 [61.0–82.0]
Sex, n (%)		
Female	39 (65.0)	38 (63.3)
Male	21 (35.0)	22 (36.7)
Vaccinated for influenza the previous year (2014), n (%)	4 (6.7)	30 (50.0)

The efficacy and safety of a single intramuscular dose of the 2015 Southern Hemisphere split-virion trivalent inactivated influenza vaccine was assessed in an open-label trial conducted at three sites in France between May 21, 2015 and June 24, 2015 (EudraCT no. 2014-005078-12). Each 0.5-mL dose contained 15 μg of hemagglutinin per strain of A/California/7/2009 (H1N1), and A/South/Australia/55/2014 (H3N2), and B/Phuket/3073/2013 (B Yamagata lineage). Participants could not have received a vaccination for seasonal influenza within the previous 6 months as part of an annual influenza vaccination campaign or within the previous 12 months as part of a clinical trial. Further details of the vaccine composition, exclusion criteria, and study ethics are provided in the Supplemental Online Information.

Between baseline and day 21 after vaccination, hemagglutination inhibition (HAI) titers for each strain in IIV3 increased by at least 11-fold for younger adults and at least 5-fold for older adults (Table 2). After vaccination, 89%–100% of the younger adult participants and 90%–98% of the older adult participants attained seroprotection (HAI titer ≥ 40) for each strain. Also, 66%–81% of younger adults and 45%–63% of older adults seroconverted or had a significant increase in HAI titer for each strain. The lower immunogenicity in older adults was likely due to immunosenescence,^12^ combined with a higher frequency of chronic medical conditions. Regardless, post-vaccination immune responses for each strain met the former CHMP criteria for both age groups.

**Table 2.** Serum HAI antibody titers and comparison with the criteria in the former CHMP Note for Guidance.^a^

		18–60 y				≥ 60 y			
		CHMP	A/H1N1	A/H3N2	B	CHMP	A/H1N1	A/H3N2	B
Measure	Day	criterion^a^	N = 59	N = 59	N = 59	criterion^a^	N = 60	N = 60	N = 60
HAI GMT (95% CI)	0	–	52.7 (34.1, 81.4)	14.2 (10.3, 19.7)	91.6 (58.1, 144)	–	34.8 (22.5, 53.8)	22.6 (15.2, 33.6)	85.2 (55.8, 130)
	21	–	644 (470, 881)	275 (188, 401)	1012 (792, 1294)	–	269 (193, 376)	219 (146, 326)	455 (329, 630)
GMTR (95% CI)	0/21	> 2.5	12.2 (7.86, 19.0)	19.3 (12.8, 29.1)	11.1 (6.93, 17.6)	> 2	7.73 (4.95, 12.1)	9.68 (6.16, 15.2)	5.34 (3.32, 8.58)
Seroprotection^b^, % (95% CI)	0	–	64.4 (50.9; 76.4)	30.5 (19.2, 43.9)	69.5 (56.1, 80.8)		46.7 (33.7, 60.0)	36.7 (24.6, 50.1)	68.3 (55.0, 79.7)
	21	> 70%	96.6 (88.3, 99.6)	89.8 (79.2, 96.2)	100.0 (93.9, 100.0)	> 60%	93.3 (83.8, 98.2)	90.0 (79.5, 96.2)	98.3 (91.1, 100.0)
Seroconversion or significant increase^c^, % (95% CI)	0/21	> 40%	74.6 (61.6, 85.0)	81.4 (69.1, 90.3)	66.1 (52.6; 77.9)	> 30%	48.3 (35.2, 61.6)	63.3 (49.9, 75.4)	45.0 (32.1, 58.4)

Blood was collected before vaccination (day 0) and 21 days after vaccination. Serum HAI titers were measured in all vaccinated subjects with data available and as described previously.^21^ HAI titers under the lower limit of quantitation (10) were assigned a value of 5 and all HAI titers above the upper limit of quantitation (10,240) were assigned a value of 10,240. To calculate GMTs, the means and 95% CIs were determined from log_10_-transformed data using Student's *t*-distribution with n−1 degrees of freedom, after which antilog transformations were applied to the results of calculations. Abbreviations: CHMP, Committee for Human Medicinal Products; CI, confidence interval; GMT, geometric mean titer; GMTR, geometric mean of the individual ratios of the post-vaccination (day 21) HAI titer divided by the pre-vaccination (day 0) HAI titer; HAI, hemagglutination inhibition.

a Note for Guidance on Harmonization of Requirements for Influenza Vaccines (CPMP/BWP/214/96).^10^

b Seroprotection was defined as a HAI titer ≥ 40.

c Seroconversion was defined as a pre-vaccination (day 0) HAI titer < 10 and a post-vaccination (day 21) HAI titer ≥ 1:40. A significant increase was defined as a pre-vaccination HAI titer ≥ 10 and a ≥ 4-fold increase in HAI titer.

As found in other studies of IIV3,^13,14^ injection-site pain, myalgia, and headache were the most common solicited reactions in both age groups (Table 3). Most of these solicited reactions were grade 1 or 2, and all were transient. Unsolicited adverse events considered to be vaccine related but not recorded as solicited reactions were reported by two younger adults (one myalgia and one fatigue) and four older adults (two fatigue, one cough, and one sore muscles with fever), all of which were grade 1 or 2 and transient. No immediate adverse events (within 30 min of vaccination) or serious adverse events were reported. Thus, there were no new safety signals, and the vaccine was well tolerated. Although two-thirds of the participants in this study were women, which could have increased solicited reactions,^15,16^ they remained within acceptable limits.

**Table 3.** Solicited reactions.

		18–60 y	> 60 y
		(N = 59)	(N = 60)
Reaction	Maximum severity	n (%)	n (%)
Injection-site reactions within 7 days			
Pain	Any	32 (54.2)	13 (21.7)
	Grade 3	1 (1.7)	0 (0.0)
Erythema	Any	4 (6.8)	14 (23.3)
	Grade 3	0 (0.0)	1 (1.7)
Swelling	Any	3 (5.1)	4 (6.7)
	Grade 3	0 (0.0)	0 (0.0)
Induration	Any	3 (5.1)	5 (8.3)
	Grade 3	0 (0.0)	0 (0.0)
Ecchymosis	Any	1 (1.7)	0 (0.0)
	Grade 3	0 (0.0)	0 (0.0)
Systemic reactions within 7 days			
Fever	Any	0 (0.0)	0 (0.0)
	Grade 3	0 (0.0)	0 (0.0)
Headache	Any	18 (30.5)	12 (20.0)
	Grade 3	1 (1.7)	0 (0.0)
Malaise	Any	5 (8.5)	6 (10.0)
	Grade 3	0 (0.0)	0 (0.0)
Myalgia	Any	20 (33.9)	11 (18.3)
	Grade 3	1 (1.7)	0 (0.0)
Shivering	Any	8 (13.6)	3 (5.0)
	Grade 3	0 (0.0)	0 (0.0)
Solicited reactions within 3 days listed in the former CHMP Note for Guidance^a^			
Injection-site induration ≥50 mm for ≥ 4 days	–	0 (0.0)	0 (0.0)
Injection-site ecchymosis	–	2 (3.4)	1 (1.7)
Temperature >38.0°C for ≥ 24 h	–	0 (0.0)	0 (0.0)
Malaise	–	4 (6.8)	4 (6.7)
Shivering	–	7 (11.9)	3 (5.0)

Solicited reactions were collected by participants on diary cards for up to 7 days after the vaccination and were analyzed in all participants vaccinated. Erythema, swelling, induration, and ecchymosis were considered grade 1 for ≥ 25 to ≤ 50 mm, grade 2 for ≥ 51 to ≤ 100 mm, and grade 3 for > 100 mm. Fever was considered grade 1 for ≥ 38.0°C to ≤ 38.4°C, grade 2 for ≥ 38.5°C to ≤ 38.9°C, and grade 3 for ≥ 39.0°C. All other reactions were considered grade 1 for not interfering with activity, grade 2 for some interference with activity, and grade 3 for significant, preventing daily activity. Abbreviation: CHMP, Committee for Human Products for Medicinal Use.

a Note for Guidance on Harmonization of Requirements for Influenza Vaccines, CPMP/BWP/214/96.^10^

The results confirmed that the Southern Hemisphere 2015 formulation of IIV3 was well tolerated and highly immunogenic in adults and met the Brazilian regulatory authority's criteria for influenza vaccine efficacy and safety. Although the study was performed in France, the immunogenicity and tolerability of IIV3 is not expected to vary between populations and should be comparable in Brazilian adults.

In accordance with the former CHMP Note for Guidance, seroprotection was defined as a HAI titer ≥ 40.^10^ However, the revised guidelines note a lack of robust evidence supporting a correlate of protection for influenza vaccines, and seroprotection no longer needs to be evaluated to support vaccine licensure.^11^ Moreover, this historical threshold of seroprotection is losing favor as an estimate of protection.^17-20^ Indeed, immunogenicity, although obviously essential, is only one of many factors determining the real-life effectiveness of influenza vaccines.

This is one of the few reports describing the immunogenicity and safety of a Southern Hemisphere formulation of IIV3. Demonstration of the safety and high immunogenicity of IIV3 should help encourage its continued use in Brazil and other Southern Hemisphere countries.
